# FXYD3 facilitates Na^+^ and liquid absorption across human airway epithelia by increasing the transport capacity of the Na/K ATPase

**DOI:** 10.1152/ajpcell.00047.2022

**Published:** 2022-08-22

**Authors:** Camilo Cano Portillo, Raul Villacreses, Andrew L. Thurman, Alejandro A. Pezzulo, Joseph Zabner, Ian M. Thornell

**Affiliations:** Department of Internal Medicine, Pappajohn Biomedical Institute, Roy J. and Lucille A. Carver College of Medicine, University of Iowa, Iowa City, Iowa

**Keywords:** airway, epithelia, ion transport

## Abstract

Na/K ATPase activity is essential for ion transport across epithelia. FXYD3, a γ subunit of the Na/K ATPase, is expressed in the airway, but its function remains undetermined. Single-cell RNA sequencing and immunohistochemistry revealed that FXYD3 localizes within the basolateral membrane of all airway epithelial cells. To study FXYD3 function, we reduced *FXYD3* expression using siRNA. After permeabilizing the apical membrane with nystatin, epithelia pretreated with *FXYD3*-targeting siRNA had lower ouabain-sensitive short-circuit currents than control epithelia. *FXYD3*-targeting siRNA also reduced amiloride-sensitive short-circuit currents and liquid absorption across intact epithelia. These data are consistent with FXYD3 facilitating Na^+^ and liquid absorption. FXYD3 may be needed to maintain the high rates of Na^+^ and fluid absorption observed for airway and other FXYD3-expressing epithelia.

## Graphical abstract

**Figure:**
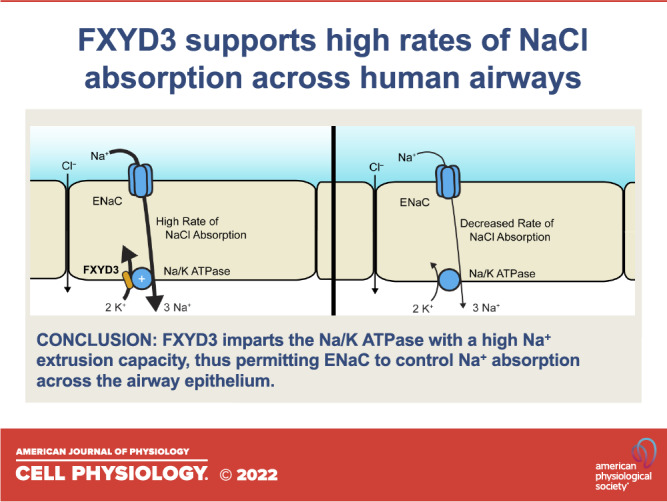

## INTRODUCTION

The Na/K ATPase maintains the Na^+^ and K^+^ gradients responsible for the potential difference across every mammalian cell membrane. With each pumping cycle, the Na/K ATPase transports three Na^+^ ions out of the cell and two K^+^ into the cell. In an epithelium, the basolateral Na/K ATPase directly participates in Na^+^ absorption. Furthermore, the high intracellular [K^+^] maintained by the Na/K ATPase establishes the electronegative Nernst potential across K^+^ channels. Thus, Na/K ATPase indirectly establishes the driving force for Cl^–^ secretion and Na^+^ absorption (1, 2).

The ion-pumping α subunit of Na/K ATPase requires a β subunit for proper trafficking to the cell membrane. Although not required for Na/K ATPase activity, FXYD proteins can bind to the Na/K ATPase complex and stimulate or inhibit its activity (3). Therefore, FXYD proteins are commonly called the γ subunit of the Na/K ATPase.

*FXYD3* mRNA transcripts are within the top 10% of transcripts expressed by human airway epithelia (4), but data are lacking for their function in epithelial cells. In humans, FXYD3 exists as two isoforms, short-FXYD3 and long-FXYD3 (5). Short-*FXYD3* contains a 78-bp deletion within the long-*FXYD3* nucleotide sequence. Both human FXYD3 isoforms stimulate Na/K ATPase activity when expressed in *Xenopus* oocytes (5). Regarding airway disease, FXYD3 expression decreases during the transition from an airway epithelial cell to a lung cancer cell (6). However, FXYD3’s contribution to airway physiology remains unstudied. Here, we used cultured human airway epithelia to investigate the expression and function of FXYD3.

## METHODS

### FXYD3 Knock-Down within Airway Epithelial Cells

Airway epithelial cells were obtained through the Iowa Donor network and studies were approved by the University of Iowa Institutional Review Board (No. 199507432). Epithelial cells were cultured according to a published protocol (7). Briefly, tissues were digested with pronase to release cells. Then, cells were seeded on collagen-coated semipermeable membranes [0.33 cm^2^ polycarbonate filters, Costar No. 3413 (Thermo Fisher Scientific)]. At the time of seeding, the cells were transfected with siRNA to FXYD3 or control siRNA (Integrated DNA Technologies, Table 1) using RNAiMAX transfection reagent (Thermo Fisher Scientific) and a previously established protocol (8).

**Table 1.** SiRNA used in this study

Target	Duplex Information
*FXYD3*	IDT# hs.Ri.FXYD3.13.1
	5′-rGrGrArCrGrCrCrArArUrGrArCrCrUrArGrA
	5′-rUrUrUrArUrCrUrUrCrUrArGrGrUrCrArUrU
*Negative Control*	IDT# DS NC 1

### Immunocytochemistry

Primary human airway epithelia were fixed with 4% paraformaldehyde and permeabilized with 0.2% Triton X-100 (Thermo Scientific). Nonspecific binding was blocked by incubating the epithelia with Superblock blocking buffer (Thermo Scientific) for 1 h. To determine the localization of FXYD3, the epithelia were incubated with three primary antibodies: 1:1,000 mouse anti-ATP1A1 (Sigma-Aldrich; 05–369-25UG, Lot 3484974), 1:50 rabbit anti-FXYD3 (Sigma-Aldrich; HPA010856; Lot A57803), and 1:50 Phalloidin conjugated with Alexa-fluor 647 (Thermo Fischer) overnight at 4°C. Then, the primary antibodies were washed, and epithelia were incubated with conjugated secondary antibodies: goat anti-mouse-Alexa-488 and goat anti-rabbit-Alexa-568, both at 1:1,000 dilution, for 1 h at room temperature protected from light. The secondary antibodies were washed, and the epithelia were mounted onto glass slides with Vectashield mounting medium with DAPI for nuclear staining (Vector Laboratories).

### RT-qPCR

Cells studied in Ussing chambers were rinsed with fresh ringer after each experiment. Inserts were then dismounted from the chamber and filters were cut and placed in Qiazol (Qiagen). RNA was isolated using the RNeasy Mini Kit (Qiagen) per manufacturer’s instructions. cDNA was synthesized from RNA using the SuperScript Vilo Mastermix kit (Invitrogen). mRNA expression was evaluated with RT-qPCR using SYBR Green chemistry and a Quant Studio 6 Real Time system. All primer pair efficiencies were confirmed by serial dilution. Primer pairs are listed in Table 2.

**Table 2.** RT-qPCR primers used in this study

Target	Forward Primer	Reverse Primer
*RPL13A*	5′-GGCCCCTACCACTTCCG	5′-ACTGCCTGGTACTTCCA
*ATP1A1*	5′-GGCAGTGTTTCAGGCTAACCAG	5′-TCTCCTTCACGGAACCACAGCA
*ATP1B1*	5′-CCCAAATGTCCTTCCCGTTCAG	5′-GCAGGAGTTTGCCATAGTACGG

### RT-PCR

The primer pair to evaluate long- versus short-*FXYD3* mRNA transcripts was designed to common nucleotides of *FXYD3* isoforms that flank the 78-bp deletion of short-*FXYD3* to yield either 97- (short) or 175-bp PCR products. The primers were forward 5′- TCATCTGCGCTGGGGTTCTGT-3′ and reverse 5′- CCTGGATGGTGACCGGACTTCTG-3′. PCR was performed using cDNA samples (obtained by methods described in *RT-qPCR*) and a Platinum TAQ polymerase kit (Life Technologies). PCR products were separated with low voltage on a 3% agarose Tris-borate-EDTA gel.

### Single-Cell RNA Sequencing

Single cell RNA-seq data from healthy human airway epithelia and lung were downloaded from Gene Expression Omnibus (GEO) via GEO accessions GSE185048 and GSE122960, respectively. In brief, gene-by-cell count matrices were normalized and scaled, followed by dimension reduction and clustering. Clusters were associated with known cell types by finding markers for each cluster and identifying cell types enriched for each set of marker genes. For a given cell type and donor, gene expression was quantified by summing up gene counts for all cells, dividing each summed gene count by total counts across all genes and cells, and scaling to counts per million. All analyses were performed using the R package Seurat version 3.1.1 (9).

### Membrane Biotinylation

To assess the expression of the Na/K ATPase at the plasma membrane, we used a protocol based on that used to biotinylate CFTR (Cystic Fibrosis Transmembrane Conductance Regulator) in airway cultures (10). All steps were performed at 4°C. After washing the epithelia three times with PBS +/+ (pH 8.2) for 2 min per wash, the cells were incubated for 60 min with 1 mg/mL biotin (PBS +/+; pH 8.2). After incubation, cells were washed three times with PBS +/+ (pH 8.2) for 2 min. Each filter containing epithelial cells was excised from the transwell and added to a lysis buffer (100 mM NaCl, 50 mM TRIS, pH 8.2, 1 µg/mL pepstatin A, 7 µg/mL benzamide, 2 µg/mL leupeptin, 2 µg/mL aprotinin, 0.01% sodium deoxycholate, and 0.2% NP40). Lysis reactions were placed on ice for 30 min and vortexed every 5 min, then each filter was removed from the lysis reaction, and the remaining lysis reaction was centrifuged (14,000 *g*; 10 min). Next, supernatants were removed, and the protein concentrations of the supernatants were obtained using a bicinchoninic acid assay kit (Pierce). Before binding the biotinylated protein to streptavidin, 100 µL of 50% streptavidin slurry was washed twice with 1 mL of PBS +/+, pH 8.2, and once with lysis buffer; centrifuging the beads (14,000 *g*, 1 min) between washes. Biotinylated protein (100 µg) was added to the streptavidin, and the remainder of the 1.5-mL microcentrifuge tube was filled with lysis buffer (without protein), which was then rotated end-over-end overnight at 4°C. The next day, biotin-streptavidin slurries were washed three times with lysis buffer and resuspended in 20-µL lysis buffer/20-µL 2× Laemmli sample buffer (4% sodium dodecyl sulfate, 100 mM dithiothreitol, 20% glycerol, 0.005% bromophenol blue, and 65 mM Tris-HCl pH 6.8). Ten micrograms of whole cell protein sample (i.e., not purified with streptavidin) were suspended in 20-µL lysis buffer/20 µL 2× Laemmli buffer. Samples were incubated at 85°C for 5 min and immediately loaded into an SDS-PAGE gel.

### Western Blot

Samples were separated by SDS-PAGE and transferred to PVDF membranes (Immobilon-FL, Millipore). The total protein for each lane was detected with revert total protein stain and washed as per manufacturer’s protocol (LI-COR). Following total protein detection, the membrane was blocked with 0.1% casein protein in PBS for 1 h at room temperature. Then, the membrane was concurrently probed with rabbit Na/K ATPase (1:1,000 dilution; Abcam; ab76020; Lot GR3184452‐8) and rabbit FXYD3 (1:250 dilution, Sigma-Aldrich; HPA010856; Lot A57803) antibodies for 2 h at room temperature. Following PBS washes, the membrane was incubated with a 1:10,000 dilution of anti-rabbit secondary antibody (LI-COR). After a final PBS wash, images were obtained with an Odyssey gel imager (LI-COR), and raw signal intensities were analyzed with ImageJ (v2.3.0/1.53f, NIH) using the approach of the NIH ImageJ user guide. We used the area under both bands observed with the Na/K ATPase for analysis because heating the sample induces Na/K ATPase dimers (11). Total protein staining was the gel loading control, and data are presented as the %*FXYD3* siRNA normalized to its donor-matched siRNA control.

### Electrophysiology

Epithelia were mounted in Ussing chambers (Physiologic Instruments). The cells were bathed in a solution containing (in mM): 135 NaCl, 2.4 K_2_HPO_4_, 0.6 KH_2_PO_4_, 1.2 CaCl_2_, 1.2 MgCl_2_, 5 dextrose, and 5 HEPES (pH = 7.4). For Cl^–^ substitution experiments, 135 mM sodium gluconate was substituted for NaCl, 1.2 mM magnesium gluconate was substituted for MgCl_2_, and 5 mM calcium gluconate was substituted for 1.2 mM CaCl_2_ (the additional Ca^2+^ compensates for Ca^2+^ chelation by gluconate). Solutions were saturated with air through a gas inlet and temperature was maintained at 37°C through a heat jacket. Voltage-sensing and current-passing electrodes containing 3 M KCl agar bridges were connected to amplifiers (VCC‐MC8, Physiologic Instruments). Transepithelial voltage (V_t_) was held at 0 mV and short-circuit current (*I*_sc_) was recorded. A 5-mV bipolar pulse was periodically applied across the epithelia to obtain the transepithelial conductance (G_t_). Drugs, from 1,000× DMSO stocks, were applied in the following order: 100 µM apical amiloride to inhibit the epithelial sodium channel (ENaC), 100 µM apical diisothiocyanostilbene-2,2′-disulfonic acid (DIDS) to inhibit non-CFTR Cl^–^ channels, 10 µM bilateral forskolin and 100 μM bilateral 3-isobutyl-1-methylxanthine (IBMX) to increase phosphokinase A-mediated CFTR activation, and 100 µM CFTR_inh_-172 to inhibit CFTR. For assessment of Na/K activity, nystatin (0.37 mg/mL) in bathing solution was added to the apical chamber, which generates current that is dependent on Na/K ATPase activity (12, 13). After a new steady-state current was established, 100 µM ouabain (from a 100 mM DMSO stock) was added to the basolateral chamber to inhibit Na/K ATPase. The effect of DMSO on short-circuit current was minimal and similar between epithelia treated with control siRNA and *FXYD3* targeting siRNA (Table 3).

**Table 3.** Changes in short-circuit current (µA cm^−2^) observed with DMSO

	% DMSO (Apical/Basolateral)			
	0.1%/0.0%	0.2%/0.0%	0.4%/0.2%	0.5%/0.2%
Control siRNA	0.39 ± 0.35	0.77 ± 0.53	1.01 ± 1.8	0.62 ± 1.39
*FXYD3* siRNA	0.60 ± 0.62	0.81 ± 0.57	0.57 ± 0.14	−0.02 ± 0.38
*P* value	0.21	0.90	0.64	0.97

Values represent the means ± SD for *n* = 4 human donors. *P* values were calculated using a paired Student’s *t* test.

### Liquid Absorption

A modified protocol was used based on study by Smith et al. (14). Cell culture media (30 µL) was added to the apical surface of culture airway epithelia and saturated mineral oil was added on top to prevent evaporation. Four hours later, liquid was retrieved with 100-µL capillary tubes (Drummond), the capillary tubes were sealed and centrifuged, then the liquid’s height within the capillary tube was measured.

## RESULTS

### FXYD3 Localizes within the Basolateral Membrane of Airway Epithelial Cells

**Figure 1.**
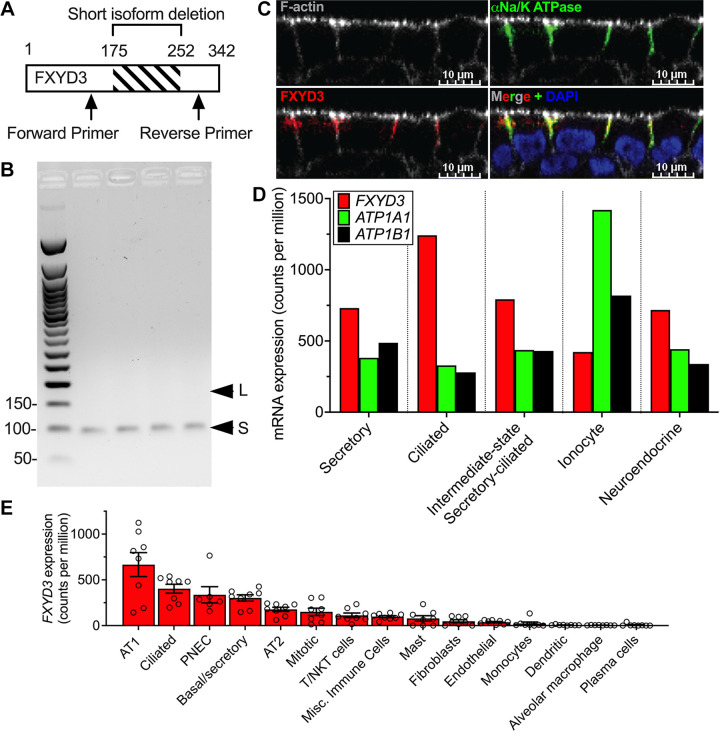
FXYD3 is expressed in human airway epithelial cells. *A*: primer design for detecting short vs. long FXYD3 isoforms. *B*: agarose gel of PCR products is shown for PCR reactions (35 cycles) containing cDNA obtained from well-differentiated airway epithelia (*n* = 4 donors); L, long-FXYD3; S, short-FXYD3. *C*: FXYD3 colocalizes with the α subunit of Na/K ATPase in the basolateral membrane of airway epithelia. Representative image from experiments performed on 5 donors. *D*: summary of single-cell RNA sequencing data of an airway epithelium cultured from cartilaginous airways. Bars represent expression level from pooled cells obtained from 5 cultures from 1 biological replicate, thus standard deviations are not shown. *E*: summary of previously published (15) single-cell RNA sequencing data of human lung performed on 8 human donors. Each dot represents 1 human donor and error bars represent the standard error of the mean. Mean counts per million ± SD; AT1 660.0 ± 369.3, Ciliated 403.2 ± 137.0, PNEC 336.8 ± 216.4, basal/secretory 302.0 ± 98.99, AT1 177.3 ± 65.92, mitotic 150.3 ± 112.7, T/NKT cells 112.5 ± 70.63, misc. immune cells 98.21 ± 32.75, mast 78.91 ± 82.96, fibroblasts 48.11 ± 45.61, endothelial 37.59 ± 20.93, monocytes 23.34 ± 44.66, dendritic 6.13 ± 5.14, alveolar macrophage 4.29 ± 2.22, plasma cells 6.48 ± 14.42. *P* values were calculated using a one-way ANOVA with Tukey correction and are presented in Table 4 for clarity. NKT, natural kill T; PNEC, pulmonary neuroendocrine cell.

FXYD3 is expressed in human airway epithelia and is absent in lung cancer cells (4, 6). However, there are no data for isoform and cellular expression in human airways. To determine the FXYD3 isoform of human airway epithelia, we performed RT-PCR using a primer pair that produces isoform-specific PCR products (Fig. 1*A*). PCR product size was consistent with short-FXYD3 expression (Fig. 1*B*). Immunocytochemistry revealed that FXYD3 colocalized with the Na/K ATPase within the basolateral membrane (Fig. 1*C*). To further identify cell types that express FXYD3, we analyzed single-cell RNA sequencing experiments performed on our cultured human epithelia. All epithelial cell types expressed *FXYD3*, *ATP1A1*, and *ATP1B1* mRNA transcripts (Fig. 1*D*). Analysis of single-cell sequencing data from human lungs (15) revealed that FXYD3 transcripts were enriched in epithelial cells versus nonepithelial cells (Fig. 1*E;* Table 4). These data suggest that short-FXYD3 regulates the Na/K ATPase activity within airway epithelial cells.

**Table 4.** P values for the Tukey-corrected ANOVA performed on tissue single-cell sequencing data

	AT1	Ciliated	PNEC	Basal/Secretory	AT2	Mitotic/Cycling	T/NKT Cells	Misc. Immune	Mast Cells	Fibroblasts	Endothelial	Monocytes	Dendritic	Alv. Macrophage	Plasma Cell
AT1	x	0.005	<0.001	<0.001	<0.001	<0.001	<0.001	<0.001	<0.001	<0.001	<0.001	<0.001	<0.001	<0.001	<0.001
Ciliated	0.005	x	>0.99	0.95	0.04	0.009	0.001	<0.001	<0.001	<0.001	<0.001	<0.001	<0.001	<0.001	<0.001
PNEC	<0.001	>0.99	x	>0.99	0.56	0.29	0.08	0.05	0.02	0.004	0.002	0.001	<0.001	<0.001	<0.001
Basal/Secretory	<0.001	0.95	>0.99	x	0.81	0.51	0.17	0.10	0.04	0.009	0.005	0.002	<0.001	<0.001	<0.001
AT2	<0.001	0.04	0.56	0.81	x	>0.99	>0.99	>0.99	0.96	0.76	0.65	0.49	0.31	0.29	0.31
Mitotic/Cycling	<0.001	0.009	0.29	0.51	>0.99	x	>0.99	>0.99	>0.99	0.95	0.90	0.78	0.60	0.58	0.60
T/NKT Cells	<0.001	0.001	0.08	0.17	>0.99	>0.99	x	>0.99	>0.99	>0.99	>0.99	0.98	0.93	0.92	0.93
Misc. Immune	<0.001	<0.001	0.05	0.10	>0.99	>0.99	>0.99	x	>0.99	>0.99	>0.99	>0.99	0.98	0.97	0.98
Mast Cells	<0.001	<0.001	0.02	0.04	0.96	>0.99	>0.99	>0.99	x	>0.99	>0.99	>0.99	>0.99	>0.99	>0.99
Fibroblasts	<0.001	<0.001	0.004	0.009	0.76	0.95	>0.99	>0.99	>0.99	x	>0.99	>0.99	>0.99	>0.99	>0.99
Endothelial	<0.001	<0.001	0.002	0.005	0.65	0.90	>0.99	>0.99	>0.99	>0.99	x	>0.99	>0.99	>0.99	>0.99
Monocytes	<0.001	<0.001	0.001	0.002	0.49	0.78	0.98	>0.99	>0.99	>0.99	>0.99	x	>0.99	>0.99	>0.99
Dendritic	<0.001	<0.001	<0.001	<0.001	0.31	0.60	0.93	0.98	>0.99	>0.99	>0.99	>0.99	x	>0.99	>0.99
Alv. Macrophage	<0.001	<0.001	<0.001	<0.001	0.29	0.58	0.92	0.97	>0.99	>0.99	>0.99	>0.99	>0.99	x	>0.99
Plasma Cells	<0.001	<0.001	<0.001	<0.001	0.31	0.60	0.93	0.98	>0.99	>0.99	>0.99	>0.99	>0.99	>0.99	X

### Short-FXYD3 Increases Airway Na/K ATPase Activity

**Figure 2.**
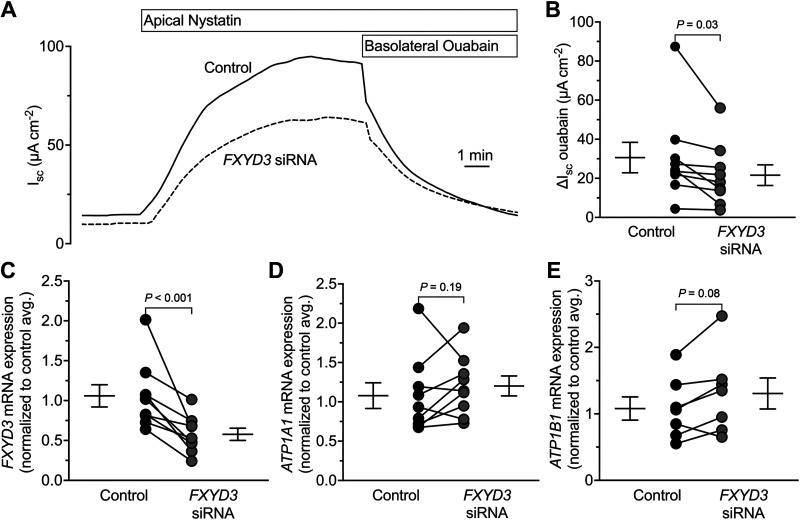
FXYD3 facilitates ion transport across the basolateral membrane of human airway epithelia. *A*: a representative short-circuit current (*I_sc_*) experiment. *B*: summary data for ouabain-sensitive *I_sc_*. Control = 30.61 ± 23.38, *FXYD3* siRNA = 21.60 ± 15.89; means ± SD. *C*: summary data for *FXYD3* expression. Control = 1.06 ± 0.42, *FXYD3* siRNA = 0.58 ± 0.23; means ± SD. *D*: summary data for *ATP1A1* expression. Control = 1.08 ± 0.49, *FXYD3* siRNA = 1.20 ± 0.38; means ± SD. *E*: summary data for *ATP1B1* expression. Control = 1.08 ± 0.46, *FXYD3* siRNA = 1.31 ± 0.46; means ± SD. For all graphs, each dot represents the average of technical replicates from 1 human donor (*n* = 9 donors) and error bars represent the standard error of the mean. *P* values were calculated using a paired Student’s *t* test. *I_sc_*, short-circuit current; SD, standard deviation.

To determine if FXYD3 affects Na/K ATPase activity, we measured the short-circuit current (*I*_sc_) across human airway epithelia pretreated with control or *FXYD3*-targeting siRNA. After mounting the epithelia, we permeabilized the apical membrane with nystatin to isolate transport across the basolateral membrane (Fig. 2*A*). The Na/K ATPase inhibitor ouabain inhibited greater *I*_sc_ for controls than epithelia pretreated with *FXYD3*-targeting siRNA (Fig. 2*B*). For these experiments, the *FXYD3*-targeting siRNA reduced *FXYD3* expression without altering *ATP1A1* and *ATP1B1* expression (Fig. 2, *C*–*E*). Therefore, FXYD3 increases Na/K ATPase activity in human airway epithelia.

**Figure 3.**
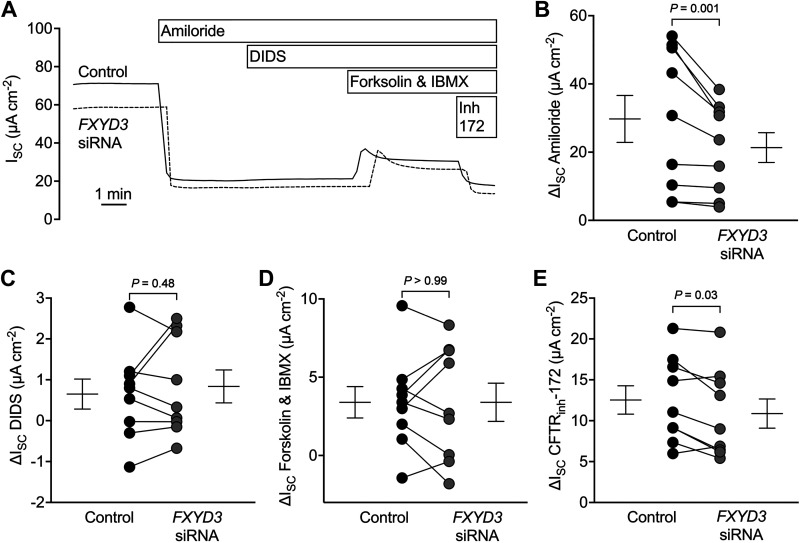
FXYD3 facilitates ion transport across human airway epithelia. *A*: a representative short-circuit current (*I_sc_*) experiment. *B*: summary data for amiloride-sensitive *I_sc_*. Control = 29.75 ± 20.66, *FXYD3* siRNA = 21.35 ± 13.10; means ± SD. *C*: summary data for DIDS-sensitive *I_sc_*. Control = 0.65 ± 1.10, *FXYD3* siRNA = 0.84 ± 1.21; means ± SD. *D*: summary data for forskolin and IBMX-sensitive *I_sc_*. Control = 3.40 ± 3.00, *FXYD3* siRNA = 3.40 ± 3.66; means ± SD. *E*: summary data for CFTR_inh_-172-sensitive *I_sc_*. Control = 12.53 ± 5.22, *FXYD3* siRNA = 10.87 ± 5.36; means ± SD. All compounds were added to the apical chamber, except for forskolin/IBMX, which was bilaterally added. For all summary data, each dot represents the average of technical replicates from 1 human donor (*n* = 9 donors) and error bars represent the standard error of the mean. *P* values were calculated using a paired Student’s *t* test. DIDS, 4,4’-diisothiocyano-2,2’-stilbenedisulfonic acid; IBMX, 3-isobutyl-1-methylxanthine; *I_sc_*, short-circuit current; SD, standard deviation.

Na/K ATPase activity directly affects apical Na^+^ absorption and indirectly affects Na^+^ and Cl^–^ transport through its influence on the K^+^ Nernst potential (1, 2). To determine whether FXYD3’s stimulation of Na/K ATPase activity affects ion transport across intact airway epithelia, we measured the *I*_sc_ for epithelia treated with control or *FXYD3*-targeting siRNA. Epithelia were sequentially exposed to amiloride, DIDS, forskolin/IBMX, and CFTR_inh_-172 (Fig. 3*A*). *FXYD3*-targeting siRNA decreased the amiloride-sensitive and CFTR_inh_-172-sensitive *I*_sc_ but did not alter DIDS-sensitive or forskolin/IBMX-stimulated *I*_sc_ (Fig. 3*B*-*D*). These data are consistent with FXYD3 facilitating Na^+^ absorption and, to a smaller extent, baseline Cl^–^ secretion.

**Figure 4.**
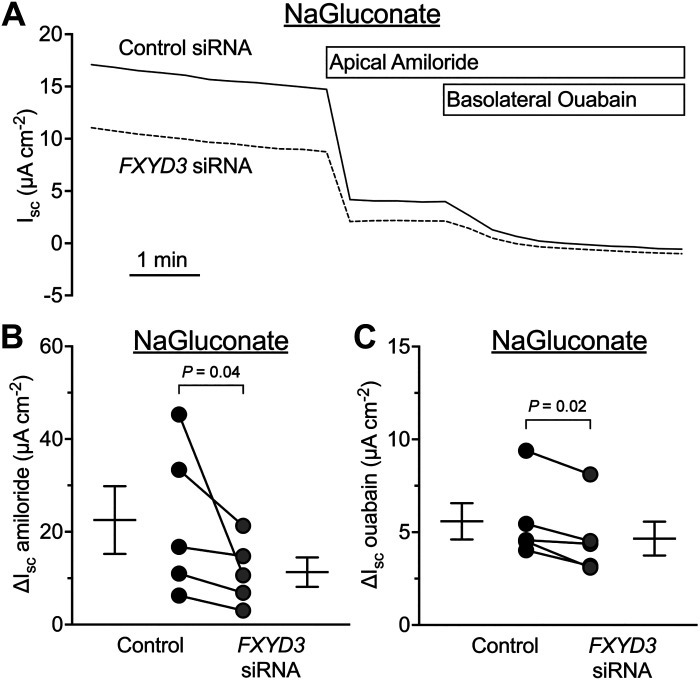
FXYD3 facilitates Na^+^ transport across human airway epithelia. *A*: a representative short-circuit current (*I_sc_*) experiment. *B*: summary data for amiloride-sensitive *I_sc_.* Control = 22.53 ± 16.34, *FXYD3* siRNA = 11.34 ± 7.08; means ± SD. *C*: summary data for ouabain-sensitive *I_sc_*. Control = 5.59 ± 2.19, *FXYD3* siRNA = 4.66 ± 2.04; means ± SD. For all summary data, each dot represents the average of technical replicates from 1 human donor (*n* = 5 donors) and error bars represent the standard error of the mean. *P* values were calculated using a paired Student’s *t* test. *I_sc_*, short-circuit current; SD, standard deviation.

Amiloride-sensitive *I*_sc_ can be affected by anion permeability (16). To evaluate FXYD3’s influence on Na^+^ absorption, we substituted gluconate for Cl^–^ in transepithelial voltage-clamp experiments (Fig. 4*A*). *FXYD3*-targeting siRNA reduced the amiloride-sensitive current and the residual ouabain-sensitive current (Fig. 4, *B* and *C*). Therefore, FXYD3 expression facilitates Na^+^ absorption.

### Short-FXYD3 Does Not Affect the Membrane Expression of Na/K ATPase

**Figure 5.**
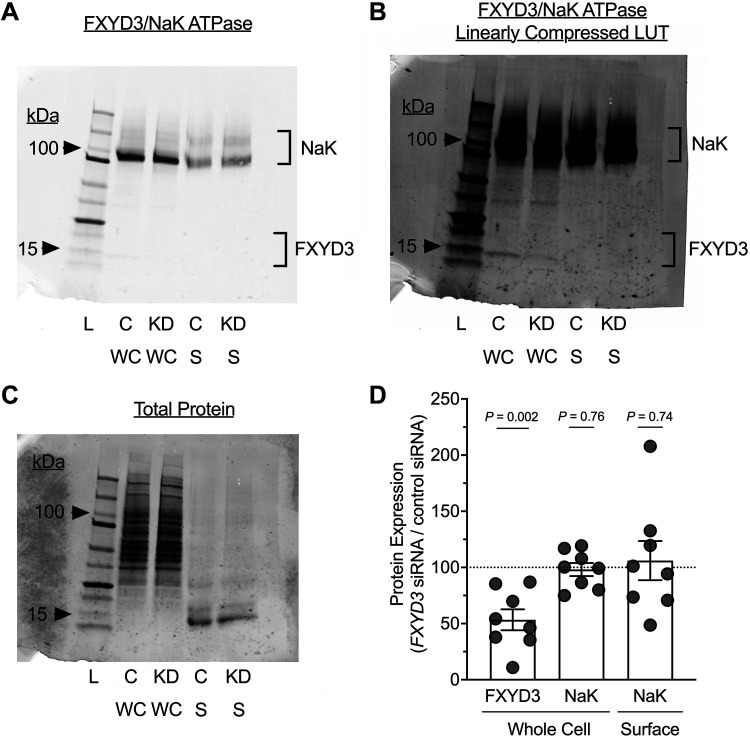
Na/K ATPase abundance in the plasma membrane of airway epithelia is unaffected by FXYD3 knockdown. *A*: a representative Western blot probed with Na/K ATPase and FXYD3 antibodies for airway epithelia from a single human donor. Lanes 2 and 3 contain whole cell protein, and lanes 4 and 5 contain biotinylated surface protein after streptavidin purification. *B*: the blot shown in *A* with the LUT linearly compressed displays the FXYD3 bands. *C*: total protein stain (loading control) of the blot shown in *A* and *B*. *D*: densitometry of raw Western blot intensities. Each donor’s *FXYD3* siRNA condition is shown as a % of the donor’s siRNA control condition. Whole cell FXYD3 = 53.35 ± 26.30, whole cell Na/K ATPase = 98.13 ± 16.50, surface Na/K ATPase = 106.0 ± 49.31; means ± SD. FXYD3 bands were below the limit of detection for streptavidin-purified samples (i.e., FXYD3 surface), thus are not reported. For all summary data, each dot represents a blot from 1 human donor (*n* = 8 donors) and error bars represent the standard error of the mean. *P* values were calculated using a one-sample *t* test with a hypothetical mean of 100% (dotted line). Precision Plus Protein Dual Color (Bio-Rad) was used as a molecular weight marker in all blots. C, siRNA control; KD, knockdown (*FXYD3* siRNA); L, ladder; LUT, look-up table; S, surface; SD, standard deviation; WC, whole cell.

The decreased Na/K ATPase activity observed with *FXYD3*-targeting siRNA can arise from a decreased transport rate or a decrease in Na/K ATPase abundance. We determined the amount of basolateral membrane Na/K ATPase by biotinylating the airway epithelial surface, then analyzing whole cell lysates and streptavidin-purified lysates by Western blot. *FXYD3*-targeting siRNA decreased cellular FXYD3 protein without affecting the cellular or plasma membrane expression of the Na/K ATPase (Fig. 5). These data suggest that FXYD3 affects the Na/K ATPase’s transport rate, rather than Na/K ATPase expression in the basolateral membrane.

### Short-FXYD3 Facilitates Liquid Absorption across the Airway Epithelium

**Figure 6.**
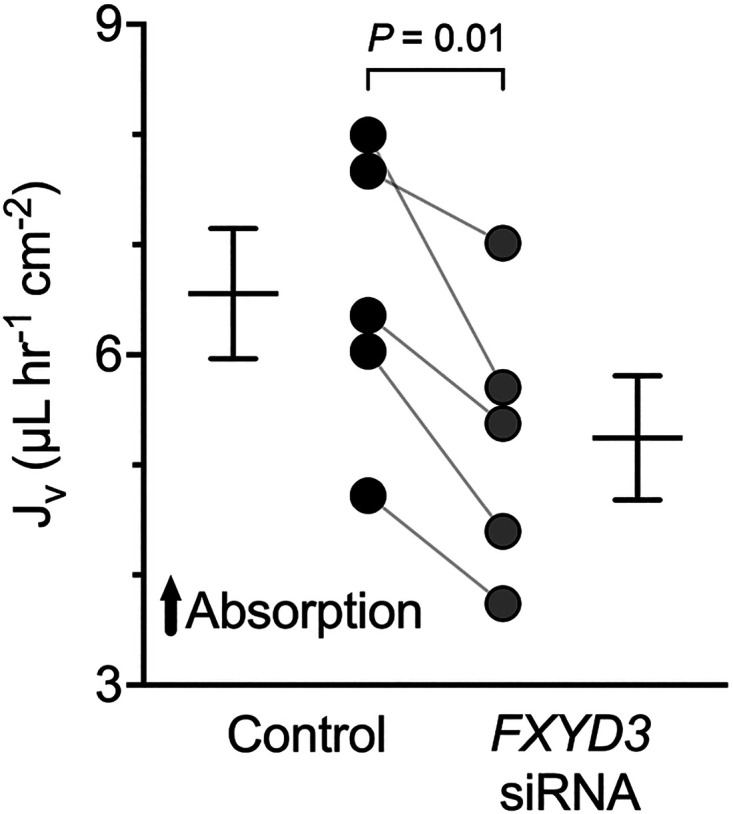
FXYD3 facilitates the rate of liquid absorption across human airway epithelia. Summary data for liquid absorption (*n* = 5 donors). Control = 6.56 ± 1.32, FXYD3 siRNA Na/K ATPase = 98.13 ± 16.50, Surface Na/K ATPase = 106.0 ± 49.31; means ± SD. Error bars represent the standard error of the mean. *P* values were calculated using Student’s paired *t* test.

Liquid transport across the airway epithelium is tightly coupled to salt transport because the epithelium is highly permeable to water (17, 18). Based on the finding that FXYD3 stimulates Na^+^ absorption, we hypothesized that FXYD3 stimulates liquid transport across the airway epithelium. We measured liquid absorption across the airway epithelium in paired control and *FXYD3*-targeting siRNA epithelia. Consistent with the finding that FXYD3 has reduced Na^+^ absorption, *FXYD3*-targeting siRNA decreased liquid absorption (Fig. 6).

## DISCUSSION

Our immunolocalization and electrophysiological data revealed that short-FXYD3 localized to the basolateral membrane of the airway epithelium. Single-cell RNA sequencing revealed that all epithelial cell types expressed FXYD3, whereas FXYD3 expression in the nonepithelial cells of the lung was nominal. In the airway epithelium, FXYD3 facilitated Na^+^ and liquid absorption across human trachea/bronchus epithelia.

The FXYD family of proteins is called the γ subunit of the Na/K ATPase because they can bind and affect Na/K ATPase function (19). Therefore, FXYD3 likely stimulated Na^+^ absorption through Na/K ATPase activity. Consistent with this proposed mechanism, we found that siRNA-mediated knockdown of FXYD3 decreased the ouabain-sensitive *I*_sc_ in the basolateral membrane of airway epithelia without altering Na/K ATPase expression in the cell membrane.

Why do airways express FXYD3? Our findings suggest that airway FXYD3 imparts the basolateral membrane’s Na/K ATPase with a high capacity to extrude Na^+^ from the cytosol. Therefore, in the presence of FXYD3, ENaC activity controls the rate of Na^+^ absorption across the airway epithelium. Consistent with this idea, ENaC overexpression increases Na^+^ absorption across the airway epithelium (20); FXYD3 overexpression fails to increase Na^+^ absorption across alveolar H441 cells (21); and in this study, knockdown of FXYD3 decreases Na^+^ absorption across human airway epithelia.

FXYD3 may alter posttranslational modifications to the Na/K ATPase. In *Xenopus* oocytes and myocytes, FXYD3 protects the Na/K ATPase from glutathionylation that occurs during oxidative stress (22, 23). Notably, airway epithelia produce H_2_O_2_ as a host defense mechanism (24). Therefore, the presence of FXYD3 may preserve Na^+^ absorption across an airway epithelium that produces copious H_2_O_2_. The glutathionylation mechanism, as described for oocytes and myocytes, was likely absent in our experimental conditions because membrane Na/K ATPase expression was unaffected by FXYD3 knockdown. However, this mechanism may exist under untested conditions (e.g., when the airways are challenged by bacteria).

The regulation of Na/K ATPase has been extensively studied in different kidney epithelial cell types. Surprisingly, FXYD3 is highly expressed in airway and alveolar epithelia, gastrointestinal epithelia from the gastric mucosa to distal enterocytes, pancreatic, cholangiocytes, and uroepithelial cells, but not in kidney proximal, distal, or collecting duct cells (25). This work should highlight the importance of regulating Na/K ATPase activity for fluid absorption in these tissues in health and disease.

Our study has limitations. Although unlikely, we cannot rule out that FXYD3 directly modified additional channels and transporters of the basolateral membrane that generate the driving force for *I*_sc_, such as K^+^ channels. The liquid transport and electrophysiological experiments require adding liquid to the airway surface liquid (ASL). Therefore, whether FXYD3 is regulated by molecules in native ASL was not determined. Our data suggest that FXYD3 is at least involved in returning ASL back to its original volume.

This study describes for the first time the function of FXYD3 in an intact airway epithelium. FXYD3 modifies ion transport across the airway epithelium by permitting high Na/K ATPase activity. Consequently, FXYD3 facilitates liquid absorption. FXYD3 may be required to efficiently drain liquid in proximal airways, thus maintaining ASL volume homeostasis.

## GRANTS

This project was funded by the National Institutes of Health’s National Heart, Lung, and Blood Institute (K01HL140261 to A.A.P., P01HL091842, and P01HL152960 to J.Z.), the Cystic Fibrosis Foundation (Iowa Research Development Program to A.A.P., J.Z., and I.M.T.), and the Gilead Sciences Research Scholars Program in Cystic Fibrosis (to I.M.T.).

## DISCLOSURES

No conflicts of interest, financial or otherwise, are declared by the authors.

## AUTHOR CONTRIBUTIONS

C.C.P., J.Z., and I.M.T. conceived and designed research; C.C.P., R.V., A.L.T., A.A.P., and I.M.T. performed experiments; C.C.P., R.V., A.L.T., A.A.P., and I.M.T. analyzed data; C.C.P, A.A.P., J.Z., and I.M.T. interpreted results of experiments; C.C.P., R.V., and I.M.T. prepared figures; C.C.P. and I.M.T. drafted manuscript; C.C.P., A.A.P., J.Z., and I.M.T. edited and revised manuscript; C.C.P., R.V., A.L.T., A.A.P., J.Z., and I.M.T. approved final version of manuscript.
